# Gene flow rise with habitat fragmentation in the bog fritillary butterfly (Lepidoptera: Nymphalidae)

**DOI:** 10.1186/1471-2148-8-84

**Published:** 2008-03-17

**Authors:** Gabriel Nève, Bernard Barascud, Henri Descimon, Michel Baguette

**Affiliations:** 1 Institut Méditerranéen d'Ecologie et de Paléoécologie, UMR CNRS 6116, Case 36, Université de Provence, 3 Place Victor Hugo, F-13331 Marseille Cedex 3, France; 2 2 Rue Rougemont, 13012 Marseille, France; 3 Muséum National d'Histoire Naturelle, Département d'Ecologie et de Gestion de la Biodiversité, CNRS-MNHN UMR 7179, Avenue du Petit-Château 4, F-91800 Brunoy, France

## Abstract

**Background:**

The main components of the spatial genetic structure of the populations are neighbourhood size and isolation by distance. These may be inferred from the allele frequencies across a series of populations within a region. Here, the spatial population structure of *Proclossiana eunomia *was investigated in two mountainous areas of southern Europe (Asturias, Spain and Pyrenees, France) and in two areas of intermediate elevation (Morvan, France and Ardennes, Belgium).

**Results:**

A total of eight polymorphic loci were scored by allozyme electrophoresis, revealing a higher polymorphism in the populations of southern Europe than in those of central Europe.

Isolation by distance effect was much stronger in the two mountain ranges (Pyrenees and Asturias) than in the two areas of lower elevation (Ardennes and Morvan). By contrast, the neighbourhood size estimates were smaller in the Ardennes and in the Morvan than in the two high mountain areas, indicating more common movements between neighbouring patches in the mountains than in plains.

**Conclusion:**

Short and long dispersal events are two phenomena with distinct consequences in the population genetics of natural populations. The differences in level of population differentiation within each the four regions may be explained by change in dispersal in lowland recently fragmented landscapes: on average, butterflies disperse to a shorter distance but the few ones which disperse long distance do so more efficiently. Habitat fragmentation has evolutionary consequences exceeding by far the selection of dispersal related traits: the balance between local specialisation and gene flow would be perturbed, which would modify the extent to which populations are adapted to heterogeneous environments.

## Background

Spatial models of genetic population structure generally refer either (i) to populations isolated in space, and connected by immigration and emigration [1], or to (ii) individuals distributed through space within a population [2,3]. However in numerous cases natural populations are distributed in clusters that may differ in their connectivity depending on landscape structure and configuration. Connectivity of a given landscape is species-specific, as exemplified by inter-species comparisons of dispersal within the same area (e.g. [4]). Moreover, connectivity depends on the evolutionary interaction between dispersal strategies on the one hand and landscape structure and configuration on the other hand, which is predicted to be species- and landscape-specific [5]. Such differences in dispersal ability among various landscapes are the result of selection of phenotypic traits linked to movement behaviour. This situation is nicely exemplified by species with a large distribution range, which end up being present in different habitat types. The nature and patchiness of their preferred habitat may then differ between different areas of their distribution range, which led to contrasted dispersal strategies in the various landscape types they occupied [6,7]. Habitat loss and fragmentation by human activities are another source of intraspecific dispersal variability. Man-induced patchiness of habitats modifies the balance between landscape structure and configuration on the one hand, and dispersal strategies on the other hand. Theory predicts that evolutionary changes in dispersal according to habitat fragmentation would be complex and non-linear [8], sometimes even maladaptive [9]. Empirical studies indeed document repeatedly various phenotypic changes according to habitat fragmentation at each of the three stages of the dispersal process (emigration from suitable habitats, displacement in the matrix and immigration in a new habitat)(review in [5]). However, the question remains about the evolutionary consequences of this phenotypic variation.

Here we address this question by comparing the effective dispersal among four different landscape types in the butterfly *Proclossiana eunomia*. Ecological studies previously showed that behavioural changes occurred in this species according to landscape structure, leading to a dispersal depression in fragmented landscapes [10]. In this study, we investigate whether such behavioural changes affect population genetic structures in landscapes differing in their structure and configuration.

A convenient way to describe dispersal strategies between species is to distinguish patchy populations from metapopulations [11]. However, these two cases of population structure are the extremes of a continuum, which depends on the proportion of individuals leaving their natal patch [12]. Locally, the area within which the individuals intermix freely has been defined as the genetic neighbourhood [2]. At a broader scale, if the differentiation between populations depends on the distance between them, as a result of equilibrium between migration and genetic drift, there is an isolation by distance effect [13].

Neighbourhood size and isolation by distance grasp two complementary levels of population spatial structure, as those parameters are informative about effective short- and long-distance dispersal respectively. Long-distance dispersal is inherently difficult to study, and various methods are now available, but all require large data sets, because of the rarity of long distance dispersal events [14]. Large data sets are more and more used to infer dispersal, either using classical *F*_ST _methods, based on Wright's island model, which per se are inadequate to estimate the number of migrants, as the hypothesis of the model rarely if ever holds in natural situations [15]. It is possible to lift the hypothesis of the island model, but this in turn requires data on effective population sizes of each population [16], which is very difficult to infer in field situations. Nevertheless, for comparative purposes, the island model approach remains a useful tool [15,17].

In this paper we would like (i) to compare neighbourhood size and isolation by distance effect in the butterfly *Proclossiana eunomia *in four different parts of its range, and (ii) from the resulting data compare the dispersal ability of the species in the respective parts of its range. We infer both neighbourhood size and isolation by distance effect from estimates of *F*_ST _and geographic distances between populations in two mainly natural (mountain) and two mainly man-shaped (fragmented) landscapes.

## Results

The genetic polymorphism of *P. eunomia *populations varied between the studied regions (Table 1; [see Additional file 1]). The Morvan is the region where *P. eunomia *shows the lowest number of polymorphic loci (three); this is due to the fact that the populations there originated from introduction performed in 1970 and 1974 from individuals coming from the Ardennes where only four polymorphic loci could be found [18-20]. Populations in the Asturias and the Pyrenees were the most variable ones with eight polymorphic loci.

**Table 1:** Mean allele frequencies in the four regions

	Alleles	PGI	PGM	6PGD	G6PDH	HBDH	AAT1	MPI	AK1
Asturias	A	0.573	0.676	0.983	1.000	0.295	0.727	1.000	1.000
	B	0.347	0.324	0.017	0.000	0.705	0.273	0.000	0.000
	G	0.010	0.000	0.000	0.000	0.000	0.000	0.000	0.000
	H	0.070	0.000	0.000	0.000	0.000	0.000	0.000	0.000

Pyrenees	A	0.804	0.702	0.768	0.683	1.000	0.771	0.541	0.862
	B	0.196	0.298	0.225	0.312	0.000	0.165	0.459	0.138
	C	0.000	0.000	0.007	0.006	0.000	0.065	0.000	0.000

Morvan	A	1.000	0.879	0.986	1.000	1.000	1.000	1.000	0.252
	B	0.000	0.121	0.014	0.000	0.000	0.000	0.000	0.748

Ardennes	A	0.997	0.700	0.746	1.000	1.000	1.000	1.000	0.546
	B	0.000	0.300	0.242	0.000	0.000	0.000	0.000	0.454
	C	0.003	0.000	0.013	0.000	0.000	0.000	0.000	0.000

**Figure 1:**
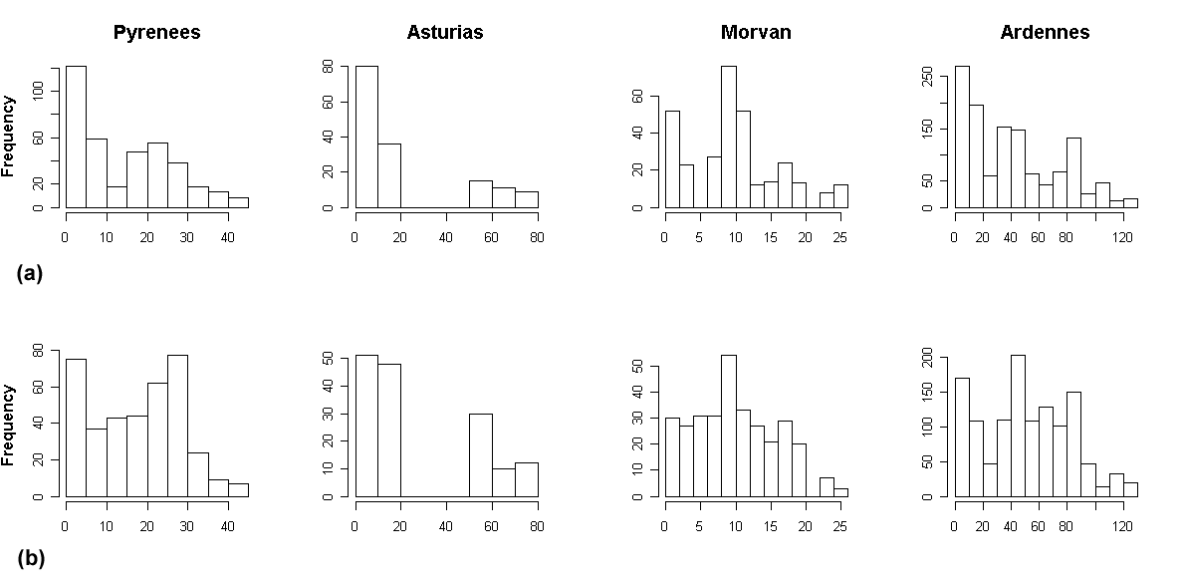
Frequencies of distances between the source and the assigned population, according to the allele frequencies. (a) Observed frequencies. (b) Frequencies of distances between the source and a randomly assigned population for each individual.

Mean *F*_ST _values per region showed that the differentiation between populations within each of the regions are moderate (sensu [2]), ranging from 0.080 for the Ardennes to 0.123 for the Asturias (Table 2). Pairwise comparisons of slopes and intercepts revealed significant differences in most cases (Table 2). The analysis of Isolation by Distance effect, with regressions of log($\hat{M}$) on log(*distance*), showed marked differences between the regions (Figure 1). In the Morvan area, the regression (and hence the slope) is not significant. In the Ardennes area, the slope is weak but significant (0.0073; [21]) whereas in the Pyrenees and the Asturias the slope was remarkably stronger (0.656 and 0.948 respectively; Table 3), indicating a stronger Isolation by distance effect in these regions (Table 1; Figure 1). The Isolation by Distance effect may then be ranked between the regions as nil in the Morvan, weak in the Ardennes, strong in the Pyrenees and strongest in the Asturias. The intercept of the log-log curves, on the other hand, indicated the reverse tendency, with the Morvan left out, as the relationship was not significant there, thus indicating an increasing neighbourhood size from the Ardennes to the Pyrenees to the Asturias.

**Table 2:** Parameters of genetic population structure within the four studied regions

	Ardennes	Morvan	Pyrenees	Asturias
Range of altitudes	280–640 m	450–870 m	1250–1900 m	1100–1250 m
Range of distances between populations	1–121 km	2–24 km	2–41 km	3–75 km
N populations	26	10	12	5
*F*_ST_	0.080	0.105	0.0985	0.123
Percentage assignment to source population	8.3%	17.6%	15.1%	40.0%
Regression of log(Nm) on log(dist)	1.305-0.0073x *P *= 0.019	NS	1.529-0.656x *P *< 0.001	1.94-0.948x *P *< 0.001
Pairwise *F*_ST _*vs *geographical distance	0.0841-0.394/(4x+1) *P* < 0.001 intercept at x = 0.92 km	NS	0.115-1.212/(4x+1) *P *= 0.009 intercept at x = 2.38 km	0.204-5.327/(4x+1) *P *= 0.0257 intercept at x = 6.27 km
Mantel test between Matrix of geographical distances and pairwise *F*_ST_	*P *= 0.019	*P *= 0.118	*P *= 0.003	*P *= 0.032
Median population connectivity (min. and max. values)	0.011 (26.2 10^-6 ^to 0.183)	0.00999 (418 10^-6 ^to 0.0247)	0.00029 (43.0 10^-6 ^to 0.0152)	0.001 (0.238 10^-6 ^to 0.00289)

NS: not significant.

**Table 3:** Comparisons of slopes and intercepts of the regression of the log(Nm) as estimated by the island model ($Nm\approx \frac{1}{4}\left(\frac{1}{F_{ST}}-1\right)$) on log(geographic distance) for the four regions considered

Comparisons	Significance of intercept differences	Significance of slope differences
Ardennes *vs*. Pyrenees	****	****
Morvan *vs *Ardennes	**	NS
Asturias *vs *Pyrenees	**	**
Asturias + Pyrenees *vs. *Ardennes + Morvan	**	**

NS: non significant; **: *P *< 0.05; ****:*P *< 0.001

The approach advocated by Porter & Geiger [22], the intercept of *F*_ST _= a+b/(4x+1), for x being the geographical distances between populations, provided the same ranking of regions (Table 2). The stress between the 2-dimensional configuration of pairwise *F*_ST _values and pairwise geographical distances between populations varied greatly between the four regions. It was the lowest for the Asturias (< 0.0001), the highest for the Morvan (27.58), with the Ardennes (0.01035) and the Pyrenees (14.09) being intermediate.

**Figure 2:**
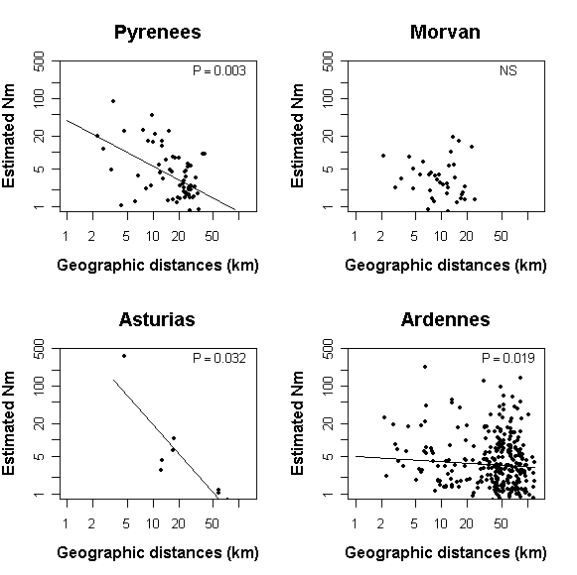
Estimated number of migrants per generation between pairs of populations (*Nm*) plotted against distances in km between populations for *P. eunomia *in the four regions studied; both with log scales. The significance levels were given by Mantel test.

The percentage of correctly assigned individuals to their population, varied between the four regions, from 8.3% in the Ardennes to 40.4% in the Asturias (Table 2). In the Ardennes, the data set with the highest number of populations (26), the procedure assigned mostly to nearby populations when the source population was not chosen as the most likely (Figure 2). As in the Morvan there is no isolation by distance (see below), the distribution of assigned individuals does not differ from the randomly assigned individuals (Wilcoxon Rank Sum Test, P = 0.95), whereas in the three other regions the assigned populations are significantly closer to the source populations than randomly assigned ones (P < 0.001).

## Discussion

Our results show that the spatial structure of *P. eunomia *varied strongly among the four studied regions. The *F*_ST _values per region all fall above the median value of *F*_ST _for European butterfly populations (reviewed in [23]). The comparison of the slope of the estimated number of effective dispersal events *vs*. geographic distance provided a ranking of the effect of isolation by distance, which was lacking in the Morvan, weak in the Ardennes and increasingly higher in the Pyrenees and the Asturias respectively; this may be related to the higher altitude range in these areas, as well as to population connectivity. The Morvan is the area with the highest recorded connectivity; it has indeed been colonised within 15 years from just two points of origin [20,24], and the populations there may not have reached an equilibrium yet (*sensu *Slatkin [13]), even if the genotypes of the individuals already show differentiation occurring within the region [19]. The slope of the pairwise log *F*_ST _*vs *log geographical distance gives an index of the Isolation by Distance effect. For the three landscapes where it could be investigated, the neighbourhood size showed a trend opposite to the one of the isolation by distance effect, being smallest in the Ardennes and largest in the Asturias, with the Pyrenees being intermediate.

Compared to high mountains, the genetic structure of *P. eunomia *populations in the lowlands of central Europe corresponds to (i) a lower dispersal rate leading to a lower neighbourhood size, and (ii) for the individuals which do disperse to a more efficient dispersal power preventing thus isolation by distance in lowland areas of relative high connectivity. The relation between dispersal ability and population genetic structure was firmly established on the basis of intrageneric but interspecific comparison [25]. At the intraspecific level, a direct relation between connectivity and gene flow was previously documented in the butterfly *Parnassius smintheus *[26]. However, here we definitely go further by showing that altogether, the pattern of genetic differentiation we observed is in line with results of mechanistic studies showing the evolution of dispersal polymorphism along a gradient of habitat fragmentation [27]. Indeed, behavioural studies demonstrated that flying individuals actively refuse to cross habitat boundaries in recently fragmented landscapes [27], which generate significantly lower dispersal emigration rates among fragmented populations [10]. This lower dispersal rate corresponds to the lower neighbourhood size among populations from lowland areas. However, individuals deciding to disperse in fragmented landscapes survive dispersal better than those dispersing in continuous landscapes [10]. This better performance has to be related to changes in the dispersal behaviour itself, dispersing butterflies in fragmented landscapes switching from routine, exploratory movements with a slow, tortuous trajectory, to special directed movements designed for net dispersal [28,29]. The absence of isolation by distance in fragmented landscapes coincides with the existence of such long-distance dispersal movements [30].

The latter point suggests that habitat fragmentation might have selected some phenotypic traits conferring better performances to dispersing individuals. Admittedly, a recent study elegantly showed that the frequency of a PGI allele was higher in more mobile *Melitatea cinxia *butterflies. Genotypes with this allele had elevated metabolic rate, which suggests selection on PGI or a closely related locus for better dispersal performance [31]. However, the main message of our paper is that changes in dispersal according to habitat fragmentation have evolutionary consequences exceeding by far such selection of dispersal related traits. We show here that isolation by distance vanished in fragmented landscapes, which corresponds to higher gene flow between local populations. Accordingly, we expect that the balance between local specialisation and gene flow should be perturbated, which would modify the extent to which populations are adapted to heterogeneous environments [32].

In lowland areas, long distance dispersal may lead to successful gene flow, as in the Ardennes the frequency of distance between populations is relatively high until 90 km. In contrast, in the Pyrenees and the Asturias, frequencies of pairwise distances drops dramatically at 30 km (Figure 2), leaving little chance to long distance dispersal, given the rarity of suitable habitat patches at that distance. Short distance dispersal, however, are favoured in these regions, leading to a higher neighbourhood size than in the Ardennes or the Morvan.

## Conclusion

**Figure 3:**
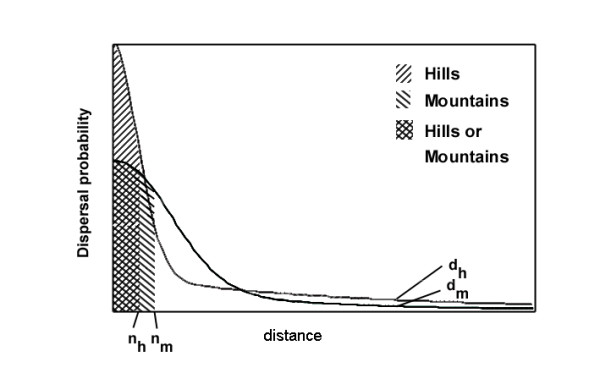
Suggested dispersal kernels in the two types of studied regions: Hills and Mountains. Shaded areas have the same probability surface between the regions. The neighbourhood size n_h_ for hill regions is smaller than the one for mountain areas, n_m_. Long distance dispersal is more frequent in hill regions (d_h_) than in mountain areas (d_m_).

The opposite trend of neighbourhood size and isolation by distance effect seems paradoxical. As individuals tend to move more, one would expect that the neighbourhood size will increase and the isolation by distance effect will get weaker. In which conditions would such opposite trends occur? Dispersal kernels do not usually follow a simple inverse exponential equation [33], the populations may be considered polymorphic as to the tendency of emigration among the individuals, and consequently the curve of the frequencies of the distances between the location of birth and the location of reproduction may be seen as the sum of curves of the different dispersal genotypes, weighted by their frequencies. Furthermore "The existence of dispersal functions valid as species-specific traits is most probably a myth [34]," so that they should be better viewed as population-specific. In the studied cases, these curves could be modelled like in figure 3. Most of the individuals tend to stay within the area classically defined as the neighbourhood size (shaded probability surface). This area is larger for the "Mountain" region (n_m_) than for the "Hill" region (n_h_). The individuals which move away from the neighbourhood area may move very far; however, their probability of leading to long distance dispersal, say of distance d, is smaller in the "Mountain" region than in the "Hill" region. Such a result may analytically be viewed as the sum of two negative exponential kernels, one for small distances, up to the range of the neighbourhood size, and another one for long distance dispersal.

Our study shows that dispersal behaviour by individuals may hardly be modelled using a single one-parameter function, however convenient this may be. For free flying insects, the factors affecting dispersal at small distances, such as mate-locating or mate-avoidance behaviour [35] or feeding behaviour, may be quite different from factors affecting long distance dispersal, such as the large scale habitat structure [36]. Furthermore, a comparison of four different habitat networks showed that the inter-patch dispersal abilities of *P. eunomia *varied according to the level of habitat fragmentation [10]. Our genetic data confirm this result and add that dispersal between neighbouring patches (i.e. short distance) may be affected differently from long-distance dispersal.

## Methods

**Figure 4:**
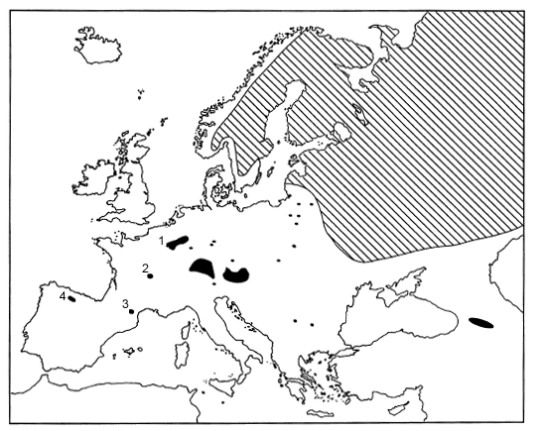
Map showing the European distribution of *P. eunomia *(from [24, 48]), indicating the four sampled regions: (1) Ardennes, (2) Morvan, (3) Pyrenees, (4) Asturias.

Between 1990 and 1995, a total of 53 populations of *Proclossiana eunomia *were sampled using butterfly nets [37,38] in four regions: the Ardennes (N. France and S. Belgium [21]), the Morvan (central France [19]), the Pyrenees (France and Spain) and the Asturias mountains (Spain)(Figure 4). In each population at least 30 individuals were captured, and frozen at -80°C until analysis. Allozyme electrophoreses were performed on these samples; a total of 13 loci were tested, and of these eight proved polymorphic in the studied area (PGI, PGM, 6PGD, G6PDH, HBDH, AAT, MPI, AK). Microsatellites were not used, as loci usable for population genetic studies are generally few and difficult to identify in Lepidoptera [39,40]. Genotypic analysis was performed using allozyme electrophoresis, as described in [21], adapted from [41].

Within the Ardennes and the Morvan, the populations were closer to each other than in the mountain areas of the Pyrenees and the Asturias, where each population was more isolated (Figure 2). If we make the hypothesis that each population inhabits a small suitable habitat surface within an unsuitable uniform area, we may compute the connectivity of each population within each region as $S_{i}=\sum_{i\neq j}\frac{1}{{\left(1+D_{ij}\right)}^{\mathrm{\alpha \alpha \#945;}}}A_{j}^{\xi_{im}}$[10], with $A_{j}^{\xi_{im}}=1$ (habitat patches are all the same size), and *D*_*ij *_the geographic distances between population i and population j. A value of 4 for α was found to be realistic for the Ardennes populations [10]. For each region the populations showed different degrees of connectivity (Table 1).

*F *statistics were calculated with the GENEPOP software [42]. Isolation by distance effect within each region was also tested by GENEPOP using a Mantel test between the matrices of pairwise *F*_ST _between populations and geographic distances between the same locations. The strength of the effect was estimated as the slope of the regression of the log-log relationship between these variables [13]. A comparison of slopes and intercepts between regions was made using dummy variables in the REG procedure, then comparing AIC between different models [43]. The fit of the genetic differentiation matrix to the geographic distance matrix was tested using multidimensional scaling. For each of the four regions, matrices of pairwise *F*_ST _were input into a 2-dimensional model, using R's mds procedure [44]. The resulting 2-dimensional structures were then compared to the geographic structure using Kruskal's Non-metric Multidimensional Scaling.

Neighbourhood size could be estimated by two methods. (i) Slatkin [13] suggested that the intercept of the log-log relationship between estimated number of migrant ($\hat{M}$) between two populations and the geographical distance between them is related to the genetic neighbourhood. (ii) The migration between populations (*Nm*) may be estimated by the relationship $Nm\approx \frac{1}{4}\left(\frac{1}{F_{ST}}-1\right)$[13].

Neighbourhood area may then be estimated by the intercept of the function of the pairwise *F*_ST _= a+b/(4x+1), where x is the geographical distance between populations, and a and b the fitted parameter values [22].

This approach on dispersal was complemented by tests of assignments. GeneClass2 [45] was used for this purpose. The Bayesian method [46] was used to calculate the probability of individual assignment to source and non-source populations, based on allele frequencies in the original populations [47]. The distance between the source population and the population assigned with the highest probability was then compared with the distance between source populations and population assigned at random within each studied region. As assignment methods accuracy depend on the polymorphism of the studied populations, the procedure was performed first with the four loci which were polymorphic in each region (PGM, PGI, 6PGD and AK). For the Pyrenees and the Asturias datasets, the whole procedure was then repeated with the full data sets.

## List of abbreviations

*D*_ij_: geographical distance between populations i and j. $\hat{M}$: estimated number of migrants between two populations. *N*: number of individuals in one or a set of populations, *m*: proportion of migrants. Studied alleles: PGI phosphoglocose isomerase E.C.5.3.1.9, PGM phosphoglucomutase E.C.2.5.7.1, 6PGD 6-phosphoclodonate dehydrogenase E.C.1.1.1.44, G6PDH glucose 6-phosphate dehydrogenase E.C.1.1.1.49, HBDH β-hydroxybutirate dehydrogenase E.C.1.1.1.30, AAT aspartate aminotransferase E.C.2.6.1.1, MPI mannose-phosphate isomerase E.C.5.3.1.8, AK adenilate kinase E.C.2.7.4.3.

## Authors' contributions

GN and BB carried out the allozyme electrophoreses. HD, now officially retired from his professorship at the University of Provence, and MB conceived the study. All authors contributed to the field work. GN carried out the data analysis and drafted the manuscript. All authors commented the manuscript and approved the final version.

## Supplementary Material

Additional file 1 **List of sampled localities, with allele frequencies and sample size**. For each locality, the coordinates are given in latitude and longitude (Greenwich coordinates) and xy coordinates in m within each region. Click here for file
